# Cellular senescence in osteoarthritis pathology

**DOI:** 10.1111/acel.12562

**Published:** 2017-01-26

**Authors:** Kendal McCulloch, Gary J. Litherland, Taranjit Singh Rai

**Affiliations:** ^1^ Institute of Biomedical and Environmental Health ResearchUniversity of the West of ScotlandPaisleyPA1 2BEUK

**Keywords:** cellular senescence, epigenetics, osteoarthritis

## Abstract

Cellular senescence is a state of stable proliferation arrest of cells. The senescence pathway has many beneficial effects and is seen to be activated in damaged/stressed cells, as well as during embryonic development and wound healing. However, the persistence and accumulation of senescent cells in various tissues can also impair function and have been implicated in the pathogenesis of many age‐related diseases. Osteoarthritis (OA), a severely debilitating chronic condition characterized by progressive tissue remodeling and loss of joint function, is the most prevalent disease of the synovial joints, and increasing age is the primary OA risk factor. The profile of inflammatory and catabolic mediators present during the pathogenesis of OA is strikingly similar to the secretory profile observed in ‘classical’ senescent cells. During OA, chondrocytes (the sole cell type present within articular cartilage) exhibit increased levels of various senescence markers, such as senescence‐associated beta‐galactosidase (SAβGal) activity, telomere attrition, and accumulation of p16ink4a. This suggests the hypothesis that senescence of cells within joint tissues may play a pathological role in the causation of OA. In this review, we discuss the mechanisms by which senescent cells may predispose synovial joints to the development and/or progression of OA, as well as touching upon various epigenetic alterations associated with both OA and senescence.

## Introduction

### Osteoarthritis (OA)

**Figure 1:**
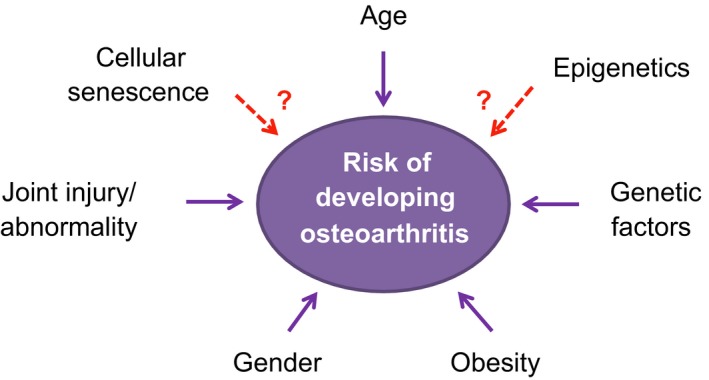
Various factors believed to contribute to the onset of OA.

Osteoarthritis (OA) is the most prevalent disease of synovial joints (around 4.7% of global population for knee and hip OA alone), afflicting many millions worldwide with pain and disability (Cross *et al*., 2014), and thus represents an enormous healthcare and socioeconomic burden. Advancing age is a major risk factor, thus the burden of OA is set to increase dramatically as populations continue to age. Gender is also recognized as a contributing factor, with the female population generally being at a higher risk of developing OA. Females are seen to develop more severe knee and hand OA compared to their male counterparts, especially when ≥55 years old (Srikanth *et al*., 2005). It has been implied that hormones may play a role in the increased incidence of OA in females, particularly a postmenopausal decrease in estrogen levels. Other risk factors contributing to the burden of OA are summarized in Fig. 1.

**Figure 2:**
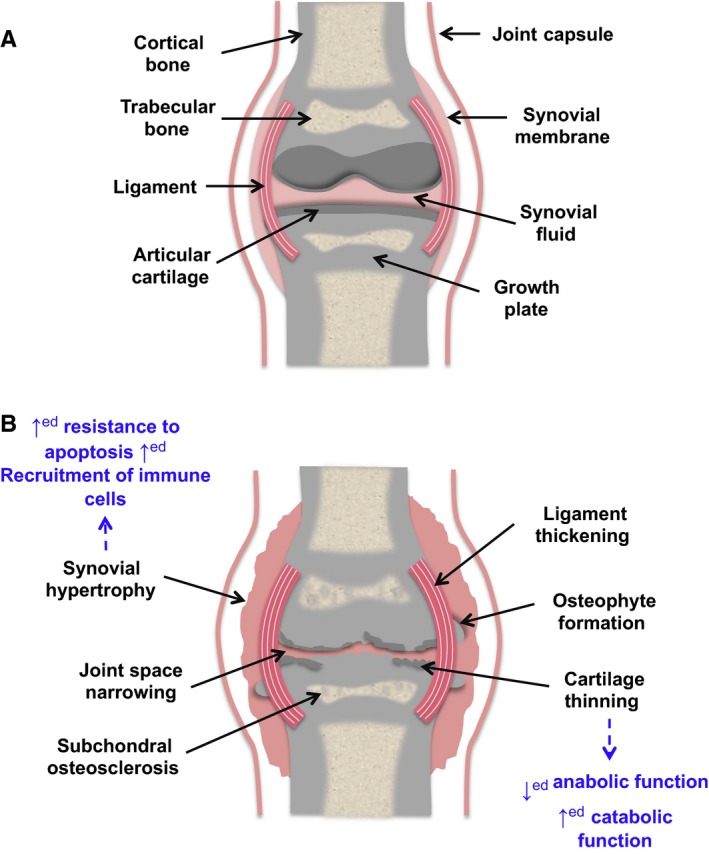
A comparison of a normal ‘healthy’ articular joint with that of a joint with OA. We also highlight the characteristics of the senescent phenotype that could potentially play a role in specific alterations seen in OA (seen in blue). (A) A diagram of a normal healthy joint. (B) A diagram of an OA‐affected joint, highlighting common changes, for example, cartilage degradation; synovial hypertrophy often accompanied by inflammation (synovitis); formation of peri‐articular osteophytes; osteosclerosis of subchondral bone.

A generalized structure for a synovial joint is illustrated in Fig. 2, characterized by the presence of connective tissues such as articular cartilage, subchondral bone, ligaments, and in some joints menisci (fibrocartilage structures that provide stability and load dispersal), encapsulated by the synovial membrane (Fig. 2A). A joint affected by OA exhibits progressive degeneration of the articular cartilage, formation of bony peripheral outgrowths (osteophytes), changes in subchondral bone and thickening of both the synovium and ligaments (Fig. 2B), and in many cases synovial inflammation (synovitis), which is thought to be an important driver of early pathology (Benito *et al*., 2005). Pathologic roles for multiple tissues in deteriorating joint function therefore define OA as a whole joint disease, driven by various biomechanical and inflammatory factors. There are currently no treatments available to effectively prevent or reverse progressive joint damage; therefore, new and innovative treatments are urgently required to improve treatment options. This will require continued improvements in our understanding of the molecular mechanisms underlying OA pathology.

### Cellular senescence

In 1961, Leonard Hayflick and Paul Moorhead first described the phenomenon known as ‘cellular senescence’, a form of ‘senescence at the cellular level’ (Hayflick & Moorhead, 1961), stating that primary human fibroblasts have a restricted lifespan of around 50 cell divisions in culture. This was once believed to be purely an *in vitro* phenomenon caused by cell culture shock; however, many research groups observed senescent cells in premalignant tissues, and this was soon discovered to be an important process *in vivo* (Dimri *et al*., 1995; Serrano *et al*., 1997; Sherr & DePinho, 2000; Michaloglou *et al*., 2005; Narita & Lowe, 2005). Cellular senescence is now considered a signal transduction process that results in cells entering a stable state of growth arrest while remaining metabolically active. Senescent cells most commonly enter this stable state in G1 phase, or early S phase, of the cell cycle (Di Leonardo *et al*., 1994; Ogryzko *et al*., 1996; Serrano *et al*., 1997; Herbig *et al*., 2004). However, senescent cells have also been observed to undergo arrest in G2 phase (Mao *et al*., 2012). Senescence ultimately results in the loss of cellular replicative capacity due to the inability of these cells to express genes required for proliferation (Dimri *et al*., 1994, 1996). Senescence is not characterized by a specific set of markers, but rather by association with a collection of cellular phenotypes that often coexist in a stressed cellular environment, such as altered morphology, chromatin structure and gene expression patterns, and an activated DNA damage response (d'Adda di Fagagna *et al*., 2003; Di Micco *et al*., 2006, 2011; d'Adda di Fagagna, 2008; Rodier *et al*., 2009). Senescent cells secrete a variety of inflammatory cytokines, growth factors and many more soluble and insoluble factors known as the senescence‐associated secretory phenotype (SASP) (Campisi, 2005), or the senescence‐messaging secretome (SMS) (Kuilman & Peeper, 2009). These factors are secreted into the cell microenvironment, with cytokines such as IL‐6 and IL‐8 enforcing the stable growth arrest of senescent cells (Acosta *et al*., 2008; Kuilman *et al*., 2008). Various features of senescent cells, such as the SASP, can cause damage to surrounding tissue (Burton *et al*., 2007). SASP secreted by senescent cells can alter the tissue microenvironment, while the senescence of stem or progenitor cells can impair tissue regeneration (Koobatian *et al*., 2015). Cells undergo senescence in response to various detrimental stimuli, including but not limited to oncogene activation; radiation; oxidative stress; shortened telomeres; and unscheduled DNA replication. Senescence is known to evoke tumor suppression, and it is widely accepted that senescence functions as a protective mechanism against cancer due to its ability to induce the proliferation arrest of damaged cells (Michaloglou *et al*., 2005; Dhomen *et al*., 2009; Goel *et al*., 2009). Aside from cancer, senescence‐associated growth arrest is also important in normal physiological processes such as wound healing (Krizhanovsky *et al*., 2008).

Over the past decade, many studies have linked cellular senescence to aging (Krishnamurthy *et al*., 2004; Baker *et al*., 2008, 2013, 2016) and age‐related pathologies (Baker *et al*., 2011), thus leading to an overlap in research between the fields of disease processes and gerontology.

### Cellular senescence and disease

In healthy individuals, the body utilizes various systems that help to prevent and/or repair cellular, molecular, and physiological damage to cells. However, these repair systems become progressively weaker during aging and the organism becomes more vulnerable to the development of a variety of diseases. Mammals such as mice, baboons, and humans have been reported to accumulate senescent cells as they age (Dimri *et al*., 1995; Krishnamurthy *et al*., 2004; Jeyapalan *et al*., 2007). Moreover, the accumulation of senescent cells in tissues contributes to both aging and the promotion of age‐related diseases (Krishnamurthy *et al*., 2004; Baker *et al*., 2008, 2016). For example, researchers have observed the presence of senescent cells (endothelial‐like cells, vascular smooth muscle cells, and macrophage‐like cells) in mice induced to develop atherosclerosis (Childs *et al*., 2016). Senescent vascular endothelial cells are present in human atherosclerotic lesions and contribute to atherogenesis (Minamino *et al*., 2002). In the context of OA, senescent cells were observed near the osteoarthritic lesions, but not in intact cartilage from the same patients and normal donors (Price *et al*., 2002; Erusalimsky & Kurz, 2005). Consistent with this, transplanted senescent cells induce an OA‐like state in mice (Xu *et al*., 2016).

We discuss below the potential mechanisms by which accumulation of senescent cells may predispose articular joints to the development and/or progression of OA. We will also discuss the role of epigenetic changes in senescent cells in the context of OA pathology and highlight potential epigenetic treatment options.

## Senescence in osteoarthritis pathology

Although there are multiple joint tissues and cell types involved in OA pathology, chondrocytes have been the focus of the vast majority of studies to date that address a role for senescence. Chondrocytes are the only cell type present in articular cartilage, a highly specialized avascular and aneural tissue whose structural and mechanical properties are largely defined by the two predominant extracellular matrix (ECM) components, type II collagen, and aggrecan. Chondrocytes are responsible for producing and maintaining this ECM and receive nutrients and external chemical signals from the synovial fluid via secretions of fibroblast‐like synoviocytes of the intimal synovial layer.

Senescent cells exhibit a SASP that enables them to communicate with other cells, as well as the microenvironment, stimulating neighboring cells to senesce (Acosta *et al*., 2013). One characteristic feature of SASP is enhanced production of vascular endothelial growth factor (VEGF), a signal protein that promotes blood vessel formation via the processes of vasculogenesis and angiogenesis. VEGF and its cognate receptors are expressed in OA cartilage and may contribute to dysregulated osteogenesis and the formation of osteophytes (Pfander *et al*., 2001; Hashimoto *et al*., 2002; Enomoto *et al*., 2003). Chondrocyte SASP is known to include production of matrix‐degrading proteases including the matrix metalloproteinases MMP‐1, and ‐13 (Philipot *et al*., 2014). MMP‐13 is thought to be central to the irreversible degradation of the cartilage type II collagen lattice in OA, partly on the basis of exogenous expression or deficiency in murine studies (Neuhold *et al*., 2001; Little *et al*., 2009).

Obesity is a major risk factor in OA, and oxidative stress resulting from excess adiposity (Keaney *et al*., 2003; Furukawa *et al*., 2004) could therefore contribute to disease partly through reactive oxygen species (ROS)‐induced pathways. Excess adiposity is also associated with an increased accumulation of senescent cells and associated SASP factors (Schafer *et al*., 2016), as proposed (Tchkonia *et al*., 2010). Further, exercise prevents diet‐induced cellular senescence as well as the SASP within visceral adipose tissue (Schafer *et al*., 2016). This suggests a possible mechanism whereby exercise‐mediated health benefits may be mediated by the prevention of senescence.

### Senescence in OA chondrocytes & cartilage

**Figure 3:**
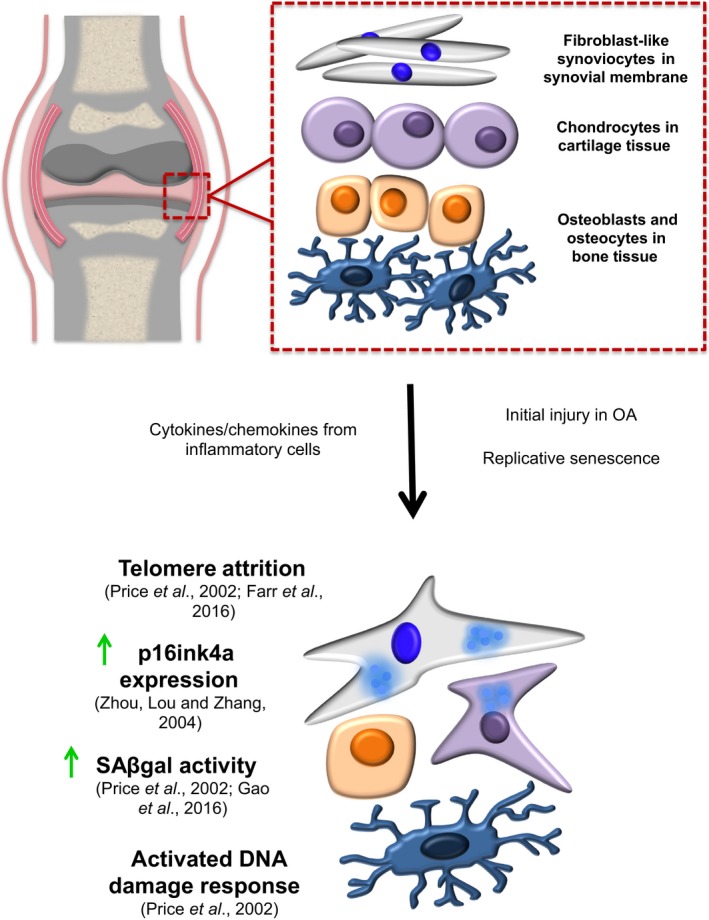
A comparison of the different characteristics observed in cell types found within joints of healthy subjects and patients with OA.

It is thought that cellular senescence may play a significant role in the pathology of OA, with OA chondrocytes exhibiting a variety of senescent‐associated phenotypes (discussed below and Fig. 3). Despite recent traction for views of OA as a whole joint disease rather than merely dysfunctional cartilage, chondrocytes remain regarded as key players in OA pathology and are understood to exhibit during disease a perturbation of the normal balance between synthesis and degradation of extracellular matrix (ECM) components. This involves upregulating the production of matrix‐degrading metalloproteinases such as MMP‐13, exogenous activity of which was sufficient to recapitulate key OA features in mice (Neuhold *et al*., 2001). Senescence of chondrocytes would be expected to lead similarly to shifting of the balance between ECM synthesis and degradation, through metalloproteinase components of the SASP response. Moreover, enhanced degradation of cartilage ECM by chondrocytes during OA may be partly due to demethylation of CpG sites in the promoter regions of genes encoding key cartilage‐degrading proteases, and therefore contributing to disease progression by increasing their production (Roach *et al*., 2005).

Aging and OA are not always interdependent, and many cases of OA in younger adults stem from joint injury (Gelber *et al*., 2000). However, as cellular senescence can result from a chronically stressed environment, it remains an interesting possibility that posttraumatic OA may be characterized or even partly triggered by an accumulation of senescent cells within damaged tissue. This view was supported, albeit *in vitro*, by the observation that mechanical stress accelerated chondrocyte senescence through increased oxidative stress (Martin *et al*., 2004a). Chondrocyte senescence is observed to be triggered by oxidative stress (Martin *et al*., 2004b) and is expected to contribute to the abnormal inflammatory environment present in OA. Chondrocytes exhibit very low metabolic activity and are well adapted to the hypoxic conditions of the joint, although exacerbated hypoxia may drive synovial inflammation in rheumatoid arthritis (RA) (an age‐related autoimmune inflammatory joint disease characterized by joint destruction, chronic inflammation, and dysfunction of innate and adaptive immune responses) contributing to pathology (Ng *et al*., 2010). As synovitis is an acknowledged feature of OA, it is plausible that enhanced hypoxia could play a similar pathologic role in this scenario (Giatromanolaki *et al*., 2003). Similarly, chronic oxidative stress experienced by other joint tissues during disease may also lead to cellular senescence.

When compared to isolated chondrocytes from normal cartilage, OA chondrocytes positively express a variety of senescence‐associated markers, for example, telomere attrition (Martin & Buckwalter, 2001); activated DNA damage response (DDR); ROS secretion; SAβGal activity (Price *et al*., 2002); increased p16ink4a expression (Zhou *et al*., 2004); accumulation of MMPs induced by pro‐inflammatory cytokines (Billinghurst *et al*., 1997; Shlopov *et al*., 1997; Fig. 3). This has led to speculation that the integrity and function of the cartilage becomes impaired due in part to the age‐related accumulation of senescent chondrocytes. In 2001, Martin and Buckwalter described telomere erosion in OA chondrocytes (Martin & Buckwalter, 2001). However, in normal articular cartilage, the rate of chondrocyte mitosis is very low (Aigner *et al*., 2001). This limited proliferative capacity in normal cartilage would suggest that other stress factors, such as oxidative stress (Yudoh *et al*., 2005) and abnormal mechanical loading (Harbo *et al*., 2012, 2013), may contribute to the shortening of telomeres of OA chondrocytes, contributing to pathology via premature senescence. Premature senescence of chondrocytes could then be a consequence of both intrinsic (limited replicative capacity *in situ*) and extrinsic (stress‐induced) factors provoking the age‐dependent deterioration of chondrocytes. On the other hand, as proliferation of chondrocytes is actually increased in OA cartilage (Aigner *et al*., 2001), this might help explain observed telomere erosion in disease. In knee OA, Gao *et al*. (2016) demonstrated a correlation between SAβGal expression and disease severity of patients. They investigated the levels of SAβGal expression in normal cartilage compared with OA cartilage of differing severity (mild, moderate, and severe). In normal articular cartilage, no staining was observed. However, in lesions taken from mild, moderate, and severely damaged knee OA cartilage, they observed SAβGal staining in a subset of chondrocytes close to the lesion. It is important to highlight that the elevation of SAβGal in cultured cells isolated from diseased joints is not a reliable indicator of pathological involvement of senescence *in vivo*. Hence, more functional experiments are required to investigate role of senescent cells in the *in vivo* development of OA.

Chondrocyte turnover is thought be a rare event in cartilage; however, these cells proliferate when removed from the tissue and placed in culture. In human OA lesions, senescent cells are often found near clusters of cells, indicating increased mitotic activity prior to senescence (Price *et al*., 2002). Further, senescent cells are known to accumulate in tissues as we age and the mere presence of them in a disease context could be a consequence of the normal aging process. For example, Martin and Buckwalter (2001) showed that an association between OA and aging is due in part to replicative senescence of chondrocytes *in vivo*. However, evidence for direct involvement of senescent cells in cartilage damage comes from the senescent cell transplantation mouse model (Xu *et al*., 2016). In a recent study, Xu *et al*. injected either senescent or nonsenescent cells into the knee joint area of mice. Authors showed that transplanting senescent cells into the knee region caused pathological features suggestive of OA (Xu *et al*., 2016). More specifically, knee joints injected with senescent cells exhibited severe articular cartilage damage at the lateral and medial tibial plateaus, as well as the femoral condyles. This would suggest targeting senescent cells might be an attractive therapeutic modality for treatment of OA. However, it is not yet fully understood how the mechanisms of chondrocyte senescence contribute to cartilage degradation, and further mechanistic studies are urgently needed.

### Senescence in the bone microenvironment

Recently senescent cells have been identified within the bone microenvironment (Farr *et al*., 2016). Comparing the presence of senescence and SASP markers in young (6 month) and old (24 month) mice, osteoblasts and osteocytes retrieved from trabecular and cortical skeletal tissue in older animals showed an increase in expression of p16Ink4a, a cell cycle inhibitor seen to increase with age (Krishnamurthy *et al*., 2004; Waaijer *et al*., 2012; Burd *et al*., 2013; Farr *et al*., 2016), concomitant with an increase in senescent osteocytes present within the bone cortex. Telomere dysfunction‐induced foci were also more prevalent in osteocytes from old mice. These findings suggest age‐related bone loss could be, in part, caused by cellular osteocyte senescence, given the vital role of these cells in bone remodeling.

### Autophagy

Autophagy is a cellular process thought to be a mechanism for cell survival when cells become stressed, for example under hypoxia or nutrient deprivation, in which cells degrade dysfunctional proteins and macromolecules and recycle them to produce the necessary raw materials for protein synthesis (Narita *et al*., 2011). There is an increasing interest in the role of autophagy in cartilage biology; this process may provide a key link between aging, cell survival, and OA. For instance, autophagy appears to be constitutively active in articular cartilage but decreases with age; an increase in apoptotic chondrocyte death was associated with a decline in autophagy and increased cartilage damage (Carames *et al*., 2010, 2015). Furthermore, chondrocyte‐specific deficiency of the autophagy factor ATG5 was recently shown to promote age‐related OA features in mice, concomitant with increased chondrocyte apoptosis (Bouderlique *et al*., 2016).

Advancing age is associated with dysregulated autophagy in cells such as cardiac myocytes (Terman *et al*., 2003); this dysregulation appears to result in oxidative stress and subsequent cellular senescence (Wu *et al*., 2009; Toshima *et al*., 2014). It is possible that these processes observed in other cell types also play a role in the cellular senescence observed in OA. Age‐related loss of skeletal muscle is a major cause of movement impairment during later stages of human life (García‐Prat *et al*., 2016). It is thought that stem cells in the muscle lose their regenerative function as we age and contribute to structural and functional decline of the muscle tissue, reviewed in detail (Grounds, 2014). In 2016, García‐Prat *et al*. (2016) described in detail the relationship between cell survival and autophagy in muscle stem/progenitor cells, with physiologically aged satellite cells undergoing senescence due to a decrease in autophagy. Young satellite cells entered senescence due to increased mitochondrial dysfunction, oxidative stress, and failure of proteostasis. By restoring the autophagy pathway in aged cells, these workers were able to show the reversal of senescence and restoration of regenerative functions (García‐Prat *et al*., 2016). Extrapolating to the context of OA, this suggests that targeting improved autophagy in joint tissues could provide a potential therapy that may lead to a decrease in inflammation, along with enhanced regeneration of joint tissues. This is a tempting idea given not only the poor regenerative capacity of articular cartilage in OA, but also with regard to senescence suppression strategies aimed at enhancing the capacity for potential use of autologous chondrocyte populations for tissue regeneration/engineering applications (Ashraf *et al*., 2016).

It is clear from the studies described above that an increased understanding of the senescent program in relation to OA would provide beneficial insight into the molecular mechanisms occurring within OA joints, which could serve to broaden the spectrum of therapeutic opportunities for the treatment of this debilitating disease. Furthermore, a renewed effort to understand cellular senescence in joint tissues other than cartilage might contribute to a more holistic view of the role of this process in OA pathology.

## Epigenetic changes in OA and cellular senescence

Both OA and cellular senescence are characterized by various epigenetic changes thought to contribute to altered cellular phenotypes and disease progression. Typically, epigenetic mechanisms can be clustered into three categories: DNA methylation involving the methylation of CpG islands; histone modifications such as acetylation, methylation, ubiquitination, and phosphorylation; and regulatory micro RNAs (small noncoding sequences involved in gene expression). We have already discussed various striking similarities of senescent cells with cells found in joint tissues during OA (particularly chondrocytes). In the following section, we will discuss the role of epigenetic changes in the context of OA, highlighting the similarities with those seen in senescence.

In human OA chondrocytes, DNA demethylation of *MMP13* promoter region CpG sites is observed to enhance the expression of MMP‐13 (Bui *et al*., 2012), a key enzyme contributing to irreversible cartilage matrix destruction. Furthermore, demethylation of the promoter sites of various other matrix‐degrading enzymes such as MMP‐3, MMP‐9 and a disintegrin and metalloproteinase with thrombospondin motifs (ADAMTS)‐4 has been associated with their enhanced expression in OA (Roach *et al*., 2005). Expression of MMP‐3, an important proteolytic activator of pro‐collagenases, is extensively used as confirmation of the senescent phenotype *in vitro* and is recognized as a ‘marker’ of senescence. In fibroblasts, loss of DNA methylation activity may cause p21‐dependent cell cycle withdrawal and senescence (Young & Smith, 2001). Interestingly, chemically mediated DNA demethylation in chondrocytes induces terminal hypertrophic differentiation (Cheung *et al*., 2001), a process essential to long bone growth in development that is recapitulated during OA (von der Mark *et al*., 1992). Beyond a commonality of enhanced MMP‐13 expression (D'Angelo *et al*., 2000), it is unclear whether there is any significant relationship between this state and cellular senescence, although cellular hypertrophy has been linked to senescence in fibroblasts (Demidenko & Blagosklonny, 2008).

It is yet to be determined whether changes in DNA methylation are causative in OA or a consequence of the disease. In human mesenchymal stem cells, workers have reported (Ezura *et al*., 2009) the presence of low levels of DNA methylation at the CpG promoters of *SOX9*, whose product maintains the chondrocyte phenotype (Lefebvre *et al*., 1997) and *RUNX2,* whose product promotes chondrocyte hypertrophy (an important factor in driving cartilage destruction) and subsequent mineralization or apoptosis (von der Mark *et al*., 1992; Enomoto *et al*., 2000; Yang *et al*., 2001; Cheung *et al*., 2003; Zheng *et al*., 2003; Kamekura *et al*., 2006). While *SOX9* and *RUNX2* gene products are critical master transcriptional regulators of chondrocyte differentiation, profoundly regulating chondrogenesis and osteogenesis, the consequences of differential methylation of their gene promoters in terms of OA are as yet unclear.

Interleukin(IL)‐1β, IL‐6, and TNF‐α, all present in the SASP of senescent cells, are key pro‐inflammatory cytokines associated with OA. In addition to their confirmed role in driving destructive MMP expression, IL‐1β and TNF‐α (in synergistic combination with the IL‐6 type cytokine, oncostatin M) have been shown to stimulate changes in the methylation pattern of the *IL1B* gene (Hashimoto *et al*., 2009), leading to potent and sustained mRNA induction. This then poses an important question of how inflammatory stimuli alter the DNA methylation pattern seen in OA. Could an influx or accumulation of senescent cells and their SASP within the joints promote these epigenetic changes?

Interestingly, hypomethylation changes are also observed in senescent cells, as well as in cancer. For example, Cruickshanks *et al*. (2013) performed whole‐genome single‐nucleotide bisulfide sequencing on human replicative senescent cells and showed widespread DNA hypomethylation, as well as focal hypermethylation of senescent cells. It is well known that one of the hallmarks of cancer is an alteration in DNA methylation resulting in the destabilization of both genome integrity and function (Matsuo *et al*., 1993; de Wind *et al*., 1995). In human cancer cells, one of the first epigenetic alterations to be observed was the low level of methylation in tumors when compared to non‐neoplastic cells (Feinberg & Vogelstein, 1983). As OA is an age‐associated disease, it is tempting to speculate that these strikingly similar features of DNA methylation could reflect the involvement of senescence gene expression programs in the pathogenesis of OA.

Gaining an understanding of the pathways and mechanisms involved in epigenetic alterations, as well as investigating the effect of various epigenetic drugs in the context of OA, would provide a promising future research area with the potential of uncovering innovative therapeutic advances. Such a method of treatment could potentially revolutionize how age‐related diseases such as OA are treated and possibly even prevented in the future.

## Future perspectives

This review explores the potential of the senescent phenotype to predispose joint tissues to the development and/or progression of OA. Senescent cells have the ability to synergize with inflammation and inflammaging, already present within the OA joint microenvironment, to drive further cellular senescent conversion and compound existing age‐related tissue damage, thus exacerbating and accelerating joint destruction. During OA, chondrocytes are seen to become ‘activated’ and express a variety of senescence‐associated markers. The exact mechanism by which senescence of chondrocytes and especially other joint cells such as synovial fibroblasts, osteoblasts, and osteocytes contribute to OA is not fully understood, although the accumulation of senescent chondrocytes in the joints is widely thought to impair cartilage integrity. Activated chondrocytes secrete a potent cocktail of cartilage‐degrading MMPs into the cartilage matrix, driving the development and progression of OA (Price *et al*., 2002). A study demonstrating the presence of senescent osteoblasts and osteocytes within the bone microenvironment of aged mice suggests an interesting potential to target these cell types to delay, or even prevent, age‐related bone loss (Farr *et al*., 2016).

Given this potential to play a key role in OA pathology, innovative treatments could be developed by gaining an understanding of the underlying mechanisms by which cellular senescence contributes to OA. Recent studies have exposed numerous common characteristics between OA cartilage and preneoplastic tissues, one of the most prevalent being cellular senescence. These recent findings have inspired researchers to explore the potential of using anticancer treatments to slow or prevent the development and/or progression of OA.

### Potential innovative therapeutic approaches

Senotherapeutic agents are used to target specific properties of cellular senescence; more specifically, senolytics are used to target anti‐apoptotic mechanisms and induce cell death within senescent cells (Zhu *et al*., 2015a,b; Chang *et al*., 2016). Senolytic drugs may therefore also be potentially used to provide an innovative therapeutic approach to treatment of various conditions. Dasatinib is currently used in the treatment of cancer. It is widely accepted that cancer cells and senescent cells share common anti‐apoptotic characteristics, and the combination treatment of dasatinib and quercetin has already been observed to reduce the burden of senescent cells, as well as enhance cardiovascular function, in aged mice (Zhu *et al*., 2015b). These workers reported, in *Ercc1* ^−/∆^ mice exhibiting an accelerated aging condition, that periodic administration of dasatinib and quercetin was seen to delay bone loss and neurological dysfunction and to enhance health span (Zhu *et al*., 2015b). We would emphasize that not all senolytic compounds are anticancer and not all anticancer compounds are senolytic. Even among senolytics there seems to be cell type specific responses, for example, dasatinib is more effective in killing senescent human preadipocytes than human umbilical vein endothelial cells (HUVECs), whereas quercetin is effective in killing senescent HUVECs rather than senescent adipocytes. More mechanistic work is needed to clarify the exact relationship of senolytics to age‐related disorders such as cancer and OA.

We have reviewed a body of work that, taken together, strongly suggests that senescence could play a significant role in the pathogenesis of OA. Therefore, if dasatinib/quercetin combination therapy is effective in eliminating senescent cells, it could provide an extremely appealing therapeutic target for OA. Although OA, unlike osteoporosis, involves not loss but mostly local increases in bone synthesis, the bisphosphonate anti‐osteoporosis drug, alendronate, has been reported to ameliorate experimental OA progression (Hayami *et al*., 2004). This suggests the possibility that senolytic drugs could also be of therapeutic value in both diseases, perhaps by preventing (likely via reducing inflammatory signaling) aberrant bone metabolism.

Epigenetic alterations have cellular impacts potentially contributing to OA development and progression, such as histone modifications that regulate catabolic mediators in cartilage (Culley *et al*., 2013), and DNA methylation present in genes known to regulate cartilage development (Ezura *et al*., 2009). In RA, the effects of epigenetic drugs are already being investigated. For instance, JQ1 is a BET bromodomain inhibitor known to downregulate Myc transcription (Delmore Jake *et al*., 2011). Myc is a transcription factor known to be upregulated in various cancers as well as aiding in the growth and invasiveness of RA synovial fibroblasts (RASFs) (Pap *et al*., 2004), a key cell type mediating joint inflammation and destruction seen in RA (Bucala *et al*., 1991; Lefevre *et al*., 2009). Recently, Zhang *et al*. (2015) showed that inhibition of bromodomain‐containing protein 4 (BRD4) by JQ1 caused a reduction in the inflammatory response, joint damage, and also autoantibody production in collagen‐induced arthritis (CIA) murine models. Xiao *et al*. (2016) further highlighted the therapeutic potential of JQ1 in inflammatory arthritis, stating JQ1 decreased the proliferation of RASFs, as well as decreasing the production of various cytokines, MMPs, and synovial inflammation in CIA. These findings, although in the context of RA, provide promising experimental evidence that targeting various epigenetic alterations could be used in the future for OA treatment, especially as synovial inflammation is now recognized to be a prominent feature of OA. Further research into the effect of epigenetic alterations in OA offers the potential to uncover novel pathways whose targeting could aid in improved treatment, delayed progression, or even possible reversal of OA.

## Conclusion

The research of recent years has established that OA is not simply a passive ‘wear and tear’ disorder, but rather a complex age‐related disease involving various different effectors, ranging from inflammatory mediators to epigenetic alterations. This complexity has undoubtedly contributed to the current lack of effective treatment options for OA, with pain management and eventual joint replacement surgery a common endpoint for a large proportion of patients. In this review, we have highlighted the potential mechanisms by which senescent cells can predispose joints of the body to the development and/or progression of OA, and explored the potential of using senolytic drugs to target senescent cells present in OA. In addition, targeting the epigenetic alterations observed in OA provides a promising approach to treatment as, unlike genetic changes, epigenetic changes can be reversed. In conclusion, gaining a better understanding of molecular mechanisms by which the senescence pathway and epigenetic changes underpin OA pathogenesis could open up novel and innovative therapeutic approaches.

## Author contributions

KM and TSR came up with the ideas and the theories. KM and TSR wrote the review with the help of GJL.

## Funding

No funding information provided.

## Conflict of interest

Authors declare there is no conflict of interest.
